# Hypersensitivity Reactions to Multiple Iodinated Contrast Media

**DOI:** 10.3389/fphar.2020.575437

**Published:** 2020-09-23

**Authors:** Inmaculada Doña, Gádor Bogas, María Salas, Almudena Testera, Esther Moreno, Jose Julio Laguna, María José Torres

**Affiliations:** ^1^Allergy Unit, Hospital Regional Universitario de Málaga, Allergy Research Group, Instituto de Investigación Biomédica de Málaga-IBIMA, ARADyAL, Málaga, Spain; ^2^Allergy Unit, University Hospital of Salamanca, Instituto de Investigación Biomédica de Salamanca-IBSAL, ARADyAL, Salamanca, Spain; ^3^Allergy Unit, Allergo-Anaesthesia Unit, Hospital Central de la Cruz Roja, Faculty of Medicine, Alfonso X El Sabio University. ARADyAL, Madrid, Spain; ^4^Nanostructures for Diagnosing and Treatment of Allergic Diseases Laboratory, Andalusian Center for Nanomedicine and Biotechnology-BIONAND, Málaga, Spain; ^5^Departamento de Medicina, Facultad de Medicina, Universidad de Málaga, Málaga, Spain

**Keywords:** anaphylaxis, drug allergy, drug provocation test, exanthema, hypersensitivity, iodinated contrast media, skin test, urticaria

## Abstract

The incidence of hypersensitivity reactions (HSRs) to iodinated contrast media (ICM) has risen over last years, representing an important health problem. HSRs to ICMs are classified into immediate reactions (IRs) and non-immediate reactions (NIRs) according to if they occur within 1 h or longer after ICM administration. The diagnosis of HSRs to ICM is complex as skin test (ST) sensitivity ranges widely, and drug provocation test (DPT) protocols are heterogeneous. In this manuscript, we describe the clinical characteristics of a series of patients confirmed as HSR to ICM and the diagnosis procedure carried out, looking into those cases confirmed as HSRs to multiple ICMs. For this purpose, we prospectively evaluated patients suggestive of HSRs to ICMs and classified them as IRs or NIRs. STs were carried out using a wide panel of ICMs, and in those with a negative ST, a single-blind placebo controlled DPT was performed with the culprit. If ST or DPT were positive, then tolerance was assessed with an alternative negative ST ICM. We included 101 cases (12 IRs and 89 NIRs) confirmed as allergic. Among them, 36 (35.64%) cases were allergic to more than one ICM (8 IRs and 28 NIRs). The most common ICM involved were iomeprol and iodixanol. Although not statistically significant, the percentage of patients reporting anaphylaxis was higher in patients allergic to multiple ICMs compared with patients allergic to a single ICM (50 vs. 25%). Likewise, the percentage of positive results in STs was higher in patients allergic to multiple ICMs compared with those allergic to a single ICM (for IR 62.5 vs. 25%, p > 0.05; and for NIR, 85.71 vs. 24.59%, p < 0.000). In cases allergic to more than one ICM, DPT with negative-ST ICM was positive in more than 60% (24/36) of cases. Therefore, allergy to multiple ICMs is common, associated to severe reactions in IRs, and confirmed frequently by positive STs. The allergological work-up should include DPT not only to establish the diagnosis but also to identify safe alternative ICM, even if ICM is structurally unrelated and ST is negative. More studies are needed to clarify mechanisms underlying cross-reactivity among ICMs.

## Introduction

Over the last decade, the incidence of hypersensitivity reactions (HSRs) to iodinated contrast media (ICM) has risen in parallel with their increased usage (Brockow et al., 2005; Brockow, 2020), being estimated to occur in about 0.5–2% of patients receiving ICMs (Brockow et al., 2005). HSRs to ICMs are classified into immediate (IRs) and non-immediate reactions (NIRs) according to if they occur within one hour or within hours or even days, respectively, after administration (Brockow et al., 2005; Brockow, 2020). Reactions may vary from mild to severe, being skin the organ most frequently involved (Brockow et al., 2005; Torres et al., 2012; Salas et al., 2013; Brockow, 2020). HSRs to ICMs have traditionally been considered as non-allergic, but growing evidence points to immune mechanisms. Positive results in skin tests (STs), basophil activation tests, and specific IgE detection in IRs suggests a likely IgE-mediated mechanism (Laroche et al., 1998; Mita et al., 1998; Laroche et al., 1999; Trcka et al., 2008; Brockow et al., 2009; Pinnobphun et al., 2011; Salas et al., 2013; Steiner et al., 2016); and the analysis of skin biopsies obtained from positive-ST and -drug provocation tests (DPTs) in NIR patients, the monitorization of the immune response during the acute and resolution phases, and the proliferative response in lymphocyte transformation test supports a T cell involvement (Romano et al., 2002; Kanny et al., 2005; Lerch et al., 2007; Torres et al., 2008; Antunez et al., 2011; Torres et al., 2012).

The diagnosis of HSRs to ICMs is complex. It is based on the clinical history, STs, and DPTs, although their role has not been fully established. The diagnostic sensitivity of STs has been reported to range from less than 5% to more than 90% (Vernassiere et al., 2004; Kvedariene et al., 2006; Trcka et al., 2008; Brockow et al., 2009; Dewachter et al., 2011; Goksel et al., 2011; Torres et al., 2012; Prieto-Garcia et al., 2013; Morales-Cabeza et al., 2017), being its routine use still matter of debate (Brockow et al., 2009; Caimmi et al., 2010; Goksel et al., 2011; Prieto-Garcia et al., 2013; Yoon et al., 2015; Soria et al., 2019). DPT is considered the gold standard for diagnosing HSRs to drugs (Aberer et al., 2003), and, in the case of HSRs to ICMs, it is recommended to be performed with the ICM giving negative results in STs for confirming diagnosis or looking for a safe alternative (Rosado Ingelmo et al., 2016; Brockow, 2020). However, its use is controversial as it is a not-risk free procedure (Aberer et al., 2003) and doses administered during the allergological work-up lack of consensus, varying from 10 to 120 cc and being injected on a single day or incrementally increased over several days (Vernassiere et al., 2004; Torres et al., 2012; Prieto-Garcia et al., 2013; Salas et al., 2013; Sese et al., 2016; Lerondeau et al., 2016; Morales-Cabeza et al., 2017; Gracia-Bara et al., 2019; Soria et al., 2019; Trautmann et al., 2019).

The management of patients diagnosed as having HSRs to ICMs involves prohibiting the use of the culprit ICM and identifying non–cross-reactive agents that can be safely used by the patient (Brockow, 2020). Currently, controversies exist regarding the pattern of cross-reactivity. Frequent cross-reactions between iodixanol, iopamidol, iomeprol, iohexol, ioversol, and ioxitalamate have been described. Cross-reactivity seems to be related to the chemical structure of ICMs, as the most frequent association has been observed between iodixanol and iohexol, being iohexol the monomer of iodixanol (Vernassiere et al., 2004; Hasdenteufel et al., 2011; Torres et al., 2012; Lerondeau et al., 2016). In fact, a classification of ICMs based on the cross-reactivity between the different molecules and related to chemical structure similarities has been proposed (Lerondeau et al., 2016). However, recommending a safe alternative in patients with HSRs to ICMs is in some cases difficult and exceptionally not possible due to the high degree of cross-reactivity. In clinical studies, reactions to several ICMs have been observed (Vernassiere et al., 2004; Torres et al., 2012; Morales-Cabeza et al., 2017; Schrijvers et al., 2018; Trautmann et al., 2019), ranging widely from 14.3% (Prieto-Garcia et al., 2013) to 88% (Brockow et al., 2009).

In this manuscript, we have analyzed a population of patients with a confirmed diagnosis of HSRs to ICMs focusing on those with HSRs to multiple ICMs.

## Methods

We prospectively evaluated patients with symptoms suggestive of HSRs to ICMs referred to the Allergy Unit of the Hospital Regional Universitario of Málaga for the period of October 2005–April 2020. Patients confirmed as allergic following a stardardized procedure including clinical history, STs, and DPTs were finally included (Rosado Ingelmo et al., 2016). In those with a confirmed diagnosis of allergy to ICM, cross-reactivity with a panel of ICMs was assessed.

Patients were classified as IRs or NIRs if reactions appeared within 1 h after ICM administration or after (Demoly et al., 2014). The clinical categories included urticaria, angioedema, and anaphylaxis for IRs, and maculopapular exanthema and delayed urticaria for NIRs (Brockow et al., 2019; Brockow, 2020). Patients with severe cutaneous reactions as Stevens-Johnson syndrome, toxic epidermal necrolysis, acute generalized pustulosis, or drug reaction with eosinophilia and systemic symptoms were excluded from the study. Severity was graded: mild when no treatment was required, moderate when the patient responded to treatment and did not require hospitalization, and severe when the patient required hospitalization (Brockow et al., 2009).

The study was conducted according to the principles of the Declaration of Helsinki. All the participants were orally informed about the study and signed the corresponding informed consent.

### Skin Test

STs were carried out as described (Torres et al., 2012; Brockow et al., 2013; Salas et al., 2013; Brockow, 2020) using a battery that included the following ICMs: iomeprol (Iomeron, Rovi, Madrid, Spain), iodixanol (Visipaque, GE Healthcare Biosciences, Madrid, Spain), iobitridol (Xenetix, Guerbet, Madrid, Spain), iohexol (Omnipaque, GE Healthcare Biosciences, Madrid, Spain), iopromide (Clarograf, Bayer, Barcelona, Spain), ioversol (Optiray, Covidien, Barcelona, Spain), and ioxaglate (Hexabrix, Guerbet, Madrid, Spain). For IRs, skin prick tests (SPTs) were performed using undiluted ICM and if negative, and intradermal tests (IDTs) were performed using 10-fold dilutions, being read 20 min after testing. For NIRs, IDTs were performed using 10-fold diluted, and if negative, undiluted ICM, being read at 20 min, 1, 2, and 3 days after testing. Positive responses were considered for SPTs if a wheal larger than 3 mm surrounded by erythema appeared with a negative response to the control saline; and for IDTs, if the size of the initial wheal increased 3 mm or more in diameter, surrounded by erythema (Brockow et al., 2002).

### Drug Provocation Test

In case of negative STs, a single-blind placebo controlled DPT was performed with the ICM involved if known, as described (Aberer et al., 2003; Torres et al., 2012; Salas et al., 2013). Additionally, in patients in which the culprit ICM was unknown and in those with a positive ST or DPT, tolerance was assessed with an alternative negative-ST ICM. For IRs, ICM was administered intravenously in saline at 45-min intervals using 5, 15, 30, and 50 cc (cumulative dose 100 cc). For NIRs, this was performed in two runs sufficiently separated to detect reactions, according to the time interval between the ICM administration and the onset of the reaction reported in the clinical history. In the first run, 5, 10, and 15 cc of ICM at 1-h intervals were administered, and if no reaction occurred, in the second run, 20, 30, and 50 cc (cumulative dose of 100 cc). Concomitant medications were stopped before DPT as previously described (Aberer et al., 2003; Rosado Ingelmo et al., 2016). As prophylaxis against renal damage, DPT procedures were separated at least 1 week, renal function was checked before ICM injection, and hydration with intravenous saline solution (0.9%) was administered if needed (Rudnick et al., 2008).

DPT was considered positive if cutaneous and/or respiratory symptoms or alterations in vital signs appeared during the procedure, then it was stopped, and the symptoms were evaluated and treated. For IRs, positive response was considered if manifestations occur up to 1 h after the DPT, and for NIRs, if cutaneous eruptions with similar clinical characteristics to those with the initial reaction occurred up to 7 days after the DPT.

### Statistical Analysis

Data analysis was performed using Chi-square analysis to test differences in nominal variables between groups, Fisher test was used when there were no criteria for using Chi-square test and Mann-Whitney test was used for quantitative variables. All reported p values represented two-tailed tests, with values <0.05 considered statistically significant.

## Results

**Figure 1:**
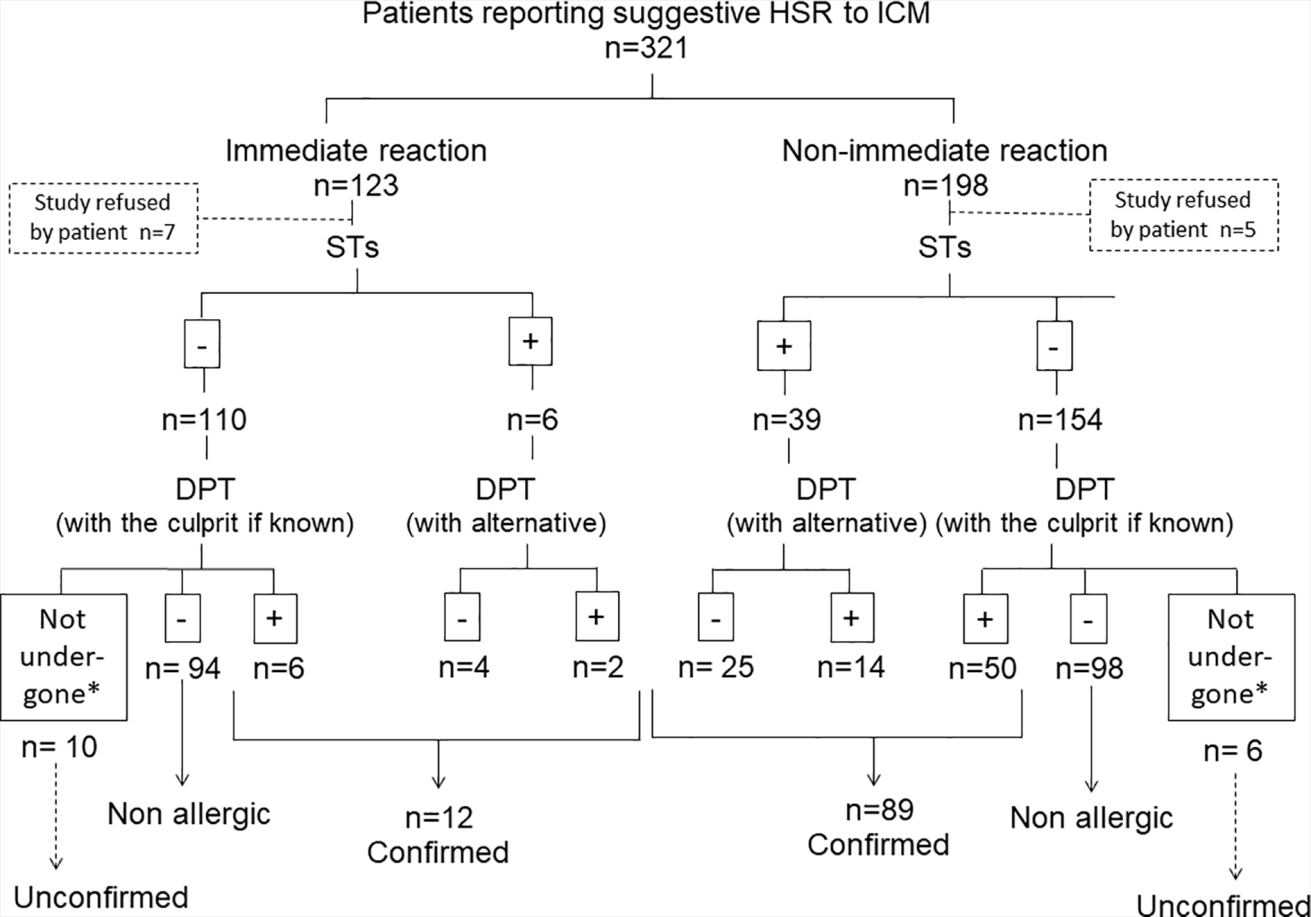
Flow-chart for the patients included in the study. *Due to contraindications for DPT: 15 cases due to comorbidities and 1 case due to the severity of the reported reaction (Stevens-Johnson syndrome).

A total of 321 subjects with a history of suggestive HSRs after at least one ICM were evaluated (106 reported IRs and 187 NIRs). From these, 220 were excluded from this study: 192 tolerated the culprit ICM (94 subjects reporting IRs and 98 NIRs) and in 28 the allergological work-up was not completed (17 IRs and 11 NIRs) due to comorbidities that contraindicated DPT (n = 15); the rejection by the patient (n = 12); and the severity of the reported reaction that contraindicated DPT (Stevens-Johnson syndrome) (n = 1). A total of 101 cases confirmed as allergic were included: 12 (11.3%) IRs and 89 (48.6%) NIRs (**Figure 1**). We included data from two previously published studies by our group that were performed in 2006–2011 (Torres et al., 2012; Salas et al., 2013).

### Clinical Characteristics and Diagnosis Approach of the Patients Included

The median age of the subjects was 62 (interquartile range: 49–69) years, and 52 (51.48%) were women. The most common ICM involved in the reactions were iodixanol (31; 30.69%) and iomeprol (33; 32.67%), followed by iohexol (16; 15.84%), iobitridol (2; 1.98%), iopramide (2; 1.98%), and ioxaglate (1; 0.99%), and in 16 (15.84%) cases, the ICM was unknown. No differences regarding age, gender, and involved ICM were found comparing IRs and NIRs. A total of 87 cases reported a single episode after ICM administration, and 14 had two episodes (all of them NIRs). Regarding the time interval between ICM administration and development of symptoms, IR patients experienced the symptoms within 1 h after administration: 9 (75%) reacted within the 30 min after the ICM administration, and 3 (25%) after 30–59 min. NIR symptoms appeared 12 h (interquartile range: 12–21) after the administration: 35 (39.32%) after 13–24 h, 34 (38.2%) after 25–48 h, 11 (12.25%) after 7–12 h, 8 (8.98%) more than 48 h later, and 1 (1.12%) after 1–6 h. According to the information obtained from the clinical history, 7 out of 12 (58.33%) cases reporting IRs developed urticaria, and 5 (41.66%) symptoms compatible with anaphylaxis. According to the severity scale of Ring and Messmer (Ring and Messmer, 1977), seven cases had grade I reactions, two had grade II reactions, and three patients had grade III reactions. No patients had grade IV reactions. Regarding NIRs, 60 (67.41%) cases had maculopapular exanthema, and 29 (32.58%) had delayed-appearing urticarial. The median time interval between the last reaction and the study was 5 months (interquartile range: 3–10). No differences were found comparing IRs and NIRs.

Regarding the results of the diagnostic methods, 6 (50%) subjects reporting IRs were diagnosed by a positive ST: 3 by SPT (1 to iodixanol, 1 to iomeprol, and 1 to iohexol) and 3 by IDT (3 to iohexol, 2 to iodixanol, 2 to iomeprol, and 1 to iobitridol). In positive-ST patients, DPT was performed with an alternative ICM, being positive in 2: one to iobitridol and one to iodixanol. In cases with a negative ST to all ICM tested, DPT was carried out with the culprit ICM if known, being positive in six cases: 4 to iomeprol, 3 to iodixanol, 2 to iobitridol, and 1 to iohexol.

Regarding NIRs, 39 (43.82%) of the subjects had a positive IDT: 24 to iomeprol, 11 to iodixanol, 7 to iohexol, 5 to iobitridol, 4 to ioxaglate, and 1 to iopramide. In positive-ST patients, DPT was performed with an alternative ICM, being positive in 14 cases: 10 to iodixanol, 4 iohexol, 4 to iobitridol, 2 to iopramide, and 1 to iomeprol. In cases with a negative ST to all ICM tested, DPT was carried out with the culprit if known, being positive in 50 cases: 41 to iodixanol, 10 to iomeprol, 4 to iobitridol, and to 4 iohexol.

Patients with positive DPT experienced similar symptoms to those recorded in their clinical history; however, they were generally milder disappearing within 1–2 h after taking corticosteroid and antihistamine drugs. Only one patient reporting IR required a dose of 0.3 cc of adrenaline by intramuscular route to resolve their reaction within one hour.

### Clinical Characteristics and Diagnosis approach of the Patients Allergic to Multiple ICMs

A total of 36 (35.64%) cases were allergic to more than one ICM, eight cases reporting IRs and 28 NIRs. This represents the 66.66% of all cases with a confirmed IR and the 31.46% of cases confirmed as NIR. The median age of the subjects was 64 (interquartile range: 49–69.5) years, and 20 (55.55%) were women. The ICMs involved in the reported reactions were iodixanol in 12 (33.33%), iomeprol in 10 (27.77%), iohexol in 7 (19.44%), ioxaglate in 1 (2.77%), and unknown in 6 (16.66%). No differences in age, gender and involved ICM were found comparing IRs and NIRs. Regarding the time interval between ICM administration and development of symptoms, IRs experienced the symptoms within 1 h after ICM administration: 7 (87.5%) cases within 30 min after the ICM administration and 1 (12.5%) with an interval if 31–59 min. NIRs appeared 10.3 (interquartile range: 6–12) h after ICM administration: 11 (39.28%) after 13–24 h, 10 (35.71%) after 25–48 h, four (14.28%) after 7–12 h, and three (10.71%) more than 48 h later. The clinical features of the reported reactions in cases allergic to multiple ICMs were urticaria in 12 (33.33%), anaphylaxis in 4 (11.11%), and MPE 20 (55.55%) (**Tables 1**, **2**).

**Table 1:** Characteristics of patients allergic to multiple ICM.

Pat	Type of reaction	Symptoms	Culprit ICM	ST							DPT				
				IOME	IOHE	IODIX	IOBIT	IOPR	IOV	IOXGL	IOME	IOHE	IODIX	IOBIT	IOPR
1	IR	ANAPH	IODIX	Neg	Pos	Pos	Neg	Neg	Neg	Neg	Neg	ND	ND	ND	ND
2	IR	ANAPH	IODIX	Pos	Pos	Pos	Neg	Neg	Neg	Neg	ND	ND	ND	Pos	Neg
3	IR	ANAPH	IOME	Pos	Neg	Neg	Neg	Neg	Neg	Neg	ND	ND	Pos	Neg	ND
4	IR	URT	IODIX	Neg	Pos	Pos	Neg	Neg	Neg	Neg	Neg	ND	ND	ND	ND
5	IR	URT	IODIX	Neg	Neg	Neg	Neg	Neg	Neg	Neg	Pos	Pos	Pos	Neg	ND
6	IR	URT	IOME	Pos	Neg	Neg	Pos	Neg	Neg	Neg	ND	Neg	ND	ND	ND
7	IR	ANAPH	UK	Neg	Neg	Neg	Neg	Neg	Neg	Neg	Pos	ND	ND	Pos	ND
8	IR	URT	IOHE	Neg	Neg	Neg	Neg	Neg	Neg	Neg	Pos	ND	Pos	Neg	ND
9	NIR	MPE	IODIX	Pos	Neg	Neg	Neg	Neg	Neg	Neg	ND	ND	Pos	ND	ND
10	NIR	MPE	IOME	Pos	Neg	Neg	Neg	Pos	Neg	Neg	ND	Neg	ND	ND	ND
11	NIR	MPE	IODIX	Neg	Pos	Pos	Neg	Neg	Neg	Neg	Neg	ND	ND	ND	ND
12	NIR	URT	IOME	Pos	Neg	Neg	Neg	Neg	Neg	Pos	ND	Neg	ND	ND	ND
13	NIR	MPE	IOME	Pos	Neg	Neg	Neg	Neg	Neg	Neg	ND	ND	Pos	ND	ND
14	NIR	MPE	IOXGL	Pos	Neg	Neg	Neg	Neg	Neg	Pos	ND	ND	Pos	Neg	ND
15	NIR	URT	IOME	Pos	Neg	Neg	Neg	Neg	Neg	Neg	ND	Pos	Pos	Neg	ND
16	NIR	URT	IOME	Pos	Neg	Neg	Neg	Neg	Neg	Neg	ND	Neg	Pos	ND	ND
17	NIR	MPE	IODIX	Neg	Pos	Pos	Neg	Neg	Neg	Neg	Neg	ND	ND	ND	ND
18	NIR	MPE	UK	Pos	Neg	Neg	Neg	Neg	Neg	Neg	ND	Pos	Pos	Neg	ND
19	NIR	URT	IODIX	Pos	Pos	Pos	Neg	Neg	Neg	Neg	ND	ND	ND	Neg	ND
20	NIR	MPE	UK	Pos	Neg	Neg	Pos	Neg	Neg	Neg	ND	ND	Neg	ND	ND
21	NIR	MPE	IOME	Pos	Neg	Neg	Neg	Neg	Neg	Neg	ND	Pos	ND	ND	ND
22	NIR	URT	IOHE	Neg	Neg	Neg	Pos	Neg	Neg	Neg	Neg	ND	ND	ND	ND
23	NIR	MPE	IOHE	Neg	Neg	Neg	Pos	Neg	Neg	Neg	Neg	ND	ND	ND	ND
24	NIR	MPE	IOHE	Pos	Neg	Neg	Neg	Neg	Neg	Neg	ND	ND	Pos	ND	ND
25	NIR	MPE	IODIX	Pos	Neg	Neg	Neg	Neg	Neg	Neg	ND	ND	Pos	ND	ND
26	NIR	URT	IOHE	Neg	Neg	Pos	Neg	Neg	Neg	Neg	Neg	Pos	ND	ND	ND
27	NIR	MPE	IODIX	Neg	Neg	Neg	Neg	Neg	Neg	Pos	ND	ND	Pos	ND	ND
28	NIR	URT	IODIX	Pos	Pos	Pos	Neg	Neg	Neg	Neg	ND	ND	ND	Pos	Pos
29	NIR	MPE	IODIX	Neg	Neg	Neg	Neg	Neg	Neg	Neg	ND	ND	Pos	Pos	ND
30	NIR	MPE	IOHE	Neg	Pos	Neg	Neg	Neg	Neg	Neg	ND	ND	Pos	Pos	ND
31	NIR	MPE	UK	Neg	Pos	Pos	Neg	Neg	Neg	Neg	Pos	ND	ND	Pos	Pos
32	NIR	URT	UK	Neg	Neg	Neg	Neg	Neg	Neg	Neg	Pos	ND	Pos	Pos	ND
33	NIR	MPE	UK	Neg	Neg	Neg	Neg	Neg	Neg	Neg	Pos	ND	ND	Pos	ND
34	NIR	MPE	IOME	Pos	Pos	Pos	Neg	Neg	Neg	Neg	ND	ND	ND	Neg	ND
35	NIR	MPE	IOME	Pos	Neg	Pos	Neg	Neg	Neg	Neg	ND	ND	ND	Pos	ND
36	NIR	MPE	IOHE	Neg	Neg	Neg	Neg	Neg	Neg	Neg	Neg	ND	ND	Pos	ND

ANAPH, anaphylaxis; DPT, drug provocation test; IOBIT, iobitridol; IODIX, iodixanol; IOHE, iohexol; IOME; iomeprol; IOPR, iopramida; IOV, ioversol; IOXGL, ioxaglate; ND, not done; Neg, negative; Pat, patient; Pos, positive; UK, unknown; URT, urticaria. ST, Skin test.

Table 2Demograhic and clinical characteristics of patients allergic to multiple ICM and those allergic to a single ICM.

**Table:** A. Immediate reactions.

		Allergic to multiple ICM n = 8	Allergic to a single ICMn = 4	p
Age; median (interquartile range) years		59 (49–65.5)	55 (38.25–62.25)	0.6278
Gender; n (%) female/n (%) male		5 (62.5)/3 (37.5)	2 (50)/2 (50)	1
Symptoms reported; n (%)	Anaphylaxis Urticaria	4 (50) 4 (50)	1 (25) 3 (75)	0.5758
ICM involved	Iodixanol Iomeprol Iohexol Iopramide Unkown	4 (50) 2 (25) 1 (12.5) – 1 (12.5)	– 1 (25) – 1 (25) 2 (50)	NA 1 NA NA 0.2364
Time interval between ICM administration and reaction onset; n (%)	≤30 min 31–59 min	7 (87.5) 1 (12.5)	2 (50) 2 50)	0.2364
N° of episodes	1 episode 2 episodes	8 (100) –	4 (100) –	1
Positive results in STs		5/8; 62.5% Iomeprol 3 Iohexol 3 Iodixanol 3 Iobitridol 1	1/4; 25 Iohexol 1	0.5455

**Table:** B. Non-immediate reactions

		Allergic to multiple ICM n = 28	Allergic to a single ICMn = 61	p
Age; median (interquartile range) years		64.5 (49–69.25)	61 (52.35–63)	0.4356
Gender; n (%) female/n (%) male		15 (53.57)/13 (42.85)	30 (49.18)/31 (50.81)	0.7004
Symptoms reported; n (%)	Urticaria MPE	8 (28.57) 20 (71.42)	21 (34.42) 40 (65.57)	0.5842
ICM involved	Iodixanol Iomeprol Iohexol Iobitridol Iopramida Ioxaglate Unkown	8 (28.57) 8 (28.57) 6 (21.42) – – 1 (3.57) 5 (17.85)	19 (27.86) 22 (32.78) 9 (13.11) 2 (3.27) 1 (1.63) – 8 (13.11)	0.8061 0.4874 0.4348 NA NA NA 0.5564
Time interval between ICM administration and reaction onset; n (%)	1–6 h 7–12 h 13–24 h 25–48 h >48 h	– 4 (14.28) 11 (39.28) 10 (35.71) 3 (10.71)	1 (1.63) – 28 (45.9) 27 (44.26) 5 (8.19)	NA NA 0.8795 0.7435 0.6998
No of episodes	1 episode 2 episodes	26 (92.85) 2 (7.14)	49 (80.32) 12 (19.67)	0.1317
Positive results in STs		24/28; 85.71% Iomeprol 16 Iohexol 7 Iodixanol 8 Iobitridol 3 Ioxaglate 3 Iopramida 1	15/61; 24.59% Iomeprol 8 Iodixanol 3 Iobitridol 2 Ioxaglate 1	0.00000006785

A.ICM, iodinated contrast media; MPE, maculopapular exanthema; NA, not applicable.

The analysis of ST results in patients with allergy to multiple ICMs showed that 5 (62.5%) cases with IRs had a positive ST: 3 by SPT (1 to iodixanol, 1 to iomeprol, and one to iohexol) and two by IDT (2 to iohexol, 2 to iodixanol, 2 to iomeprol, and 1 to iobitridol). Regarding NIRs, 24 (85.71%) subjects had a positive IDT: 16 to iomeprol, eight to iodixanol, sto iohexol, 3 to iobitridol, 3 to ioxaglate, and 1 to iopramide (**Tables 1**, **2**). DPT was performed with negative-ST ICM, being positive in 5 cases with IRs: 3 to iomeprol, 3 to iodixanol, 2 to iobitridol, and 1 to iohexol. Six cases reporting IRs were confirmed as being allergic to 2 ICMs, 1 to 3 ICMs and 1 to 4 ICMs. DPT was positive in 19 cases reporting NIRs: 12 to iodixanol, 8 to iobitridol, 4 iohexol, 3 to iomeprol, and 2 to iopramide (**Tables 1**, **3**). A total of 18 subjects reporting NIRs were confirmed as being allergic to 2 ICMs, 8 to 3 ICMs, and 2 to 5 ICMs (**Table 1**). In 14 cases, no tolerated alternative was found: 12 cases refused to perform more DPTs with others negative-ST ICMs (patients 7, 9, 13, 21, 24, 25, 27, 29, 32, 33, and 35), and 2 cases (patients 28 and 31) were confirmed to be allergic to the 5 ICMs available in our hospital (**Table 1**). The most common associations detected were iodixanol and iomeprol in 17 cases (10 by ST plus DPT, 4 by STs, and 3 by DPT) and iodixanol and iohexol in 12 cases (7 by STs, 3 by DPT, and 2 by STs plus DPT) (**Table 1**).

**Table 3:** Comparison of DPT results in patients allergic to multiple ICM.

	ICM used in DPT	DPT		p
		Positive (reacted)	Negative (tolerated)	
Total n = 36	Iomeprol Iohexol Iodixanol Iobitridol Iopramida	6 (40) 5 (50) 15 (88.23) 10 (55.55) –	9 (60) 5 (50) 2 (11.76) 8 (44.44) 3 (100)	0.1243 0.6187 0.002 0.8721 NA
IR n = 8	Iomeprol Iohexol Iodixanol Iobitridol Iopramida	3 (50) 1 (33.33) 3 (75) 2 (40) –	3 (50) 2 (66.66) 1 (25) 3 (60) 1 (100)	1 1 0.3034 1 NA
NIR n = 28	Iomeprol Iohexol Iodixanol Iobitridol Iopramida	3 (33.33) 4 (57.14) 12 (92.3) 8 (61.53) 2 (66.66)	6 (66.66) 3 (42.85) 1 (7.69) 5 (38.46) 1 (33.33)	0.05282 1 0.006 0.9877 1

DPT, drug provocation test; ICM, iodinated contrast media; NA, not applicable; IR, immediate reaction; NIR, non-immediate reaction.

### Comparison of Clinical Characteristics and Diagnosis Approach in Both Patients Allergic to Multiple ICM and Those Allergic to a Single ICM

Comparing patients allergic to more than one ICM with those allergic to a single ICM, we found that the percentage of patients reporting anaphylaxis was higher in patients allergic to multiple ICM (50 vs. 25%; p > 0.05) (**Table 2**). The percentage of cases giving positive results in STs was higher in patients allergic to multiple ICMs compared with those allergic to a single ICM in both IR and NIR groups (for IR, 62.5 vs. 25%, p > 0.05; and for NIR, 85.71 vs. 24.59%, p < 0.000), being iomeprol the most common ICM giving positive results, mainly in NIRs (**Table 2**). Iodixanol was the ICM giving most frequently positive results in DPT (p = 0.002) in both IRs and NIRs, whereas iomeprol was the most frequently tolerated ICM in DPT (p > 0.05) (**Table 3**). Although not statistically significant, patients allergic to multiple ICMs reacted in DPT to a lower dose than those cases allergic to a single ICM in both IRs [20 (20–50) vs. 35 cc [27.5–42.5), p = 0.8079) and NIRs [25 (20–82.5) vs. 50 cc (37.5–100), p = 0.1207)].

## Discussion

The incidence of HSRs to ICMs has increased over last decades (Brockow et al., 2005; Brockow, 2020), maybe due to the increase in the use of non-ionic ICMs, with approximately 75 million administrations conducted yearly worldwide (Sanchez-Borges et al., 2019). This increased incidence is a concern for doctors and patients as HSR diagnosis implies avoiding ICMs, which are required for radiological examination or treatment of different entities. The evaluation of HSRs to ICMs has been gaining attention over recent years (Brockow et al., 2005; Brockow et al., 2009; Hasdenteufel et al., 2011; Torres et al., 2012; Salas et al., 2013; Lerondeau et al., 2016; Sese et al., 2016; Soria et al., 2019; Trautmann et al., 2019; Brockow, 2020). The allergological work-up not only confirms or excludes the diagnosis but also finds safe alternative ICM. However, in some patients, finding a tolerated alternative may be difficult, as cross-reactivity among ICMs has been reported (Vernassiere et al., 2004; Kanny et al., 2005; Kvedariene et al., 2006; Brockow et al., 2009; Hasdenteufel et al., 2011; Torres et al., 2012; Salas et al., 2013; Lerondeau et al., 2016; Morales-Cabeza et al., 2017; Schrijvers et al., 2018). HSRs to multiple ICMs have been widely observed, ranging from 14% (Prieto-Garcia et al., 2013) to 88% (Brockow et al., 2009). This variability may be due to the different criteria used for patient inclusion and the different sample size in each study. In our population, 35.64% of patients were found to be allergic to two or more ICMs, being this percentage higher in IRs (66.66%) than in NIRs (31.46%). Indeed, 33.33% of our patients were allergic to three or more ICMs, and even in two patients, none of the available ICMs was tolerated. However, this percentage may be higher as in a percentage of the patients attending to our clinic because of a reaction after an ICM administration, the involved ICM was unknown, as in clinical practice, the exact name of the ICM is not always recorded in the radiologist clinical history. In these cases, as well as in those in which ICM was known but STs were negative, tolerance was assessed, and if no reaction occurs, no more ICMs are tested. This may also be the reason why the percentage of confirmed allergic patients in our population is low.

It has been considered that the diagnostic value of STs may be insufficient. A meta-analysis on STs in HSRs to ICM found an overall positive rate of STs of 17% in IRs and 26% in NIRs (Yoon et al., 2015). This may happen because the inclusion criteria are based in many cases on the clinical history. In our study, we have only included patients with a confirmed diagnosis based on STs or DPTs and in this situation 50% of IRs and 43% of NIRs gave positive results in STs. Indeed, the percentage of positive results in STs was higher in cases allergic to more than one ICM (62.5% for IR and 85.71% for NIR). It is not known the reason for this observation. For IRs, it has been reported that positive STs are associated to severity reaction (Salas et al., 2013; Yoon et al., 2015; Trautmann et al., 2019). In our study, the percentage of patients reporting severe reactions (anaphylaxis) was higher in the group of patients allergic to multiple ICMs compared with those allergic to a single ICM, although this difference was not statistically significant, probably due to the small sample size. Moreover, the time interval between the reaction and the study may also influence in having positive results in STs (Salas et al., 2013; Yoon et al., 2015), however in our study no differences were found comparing patients allergic to multiple and to a single ICM. Another factor that must to be taken into account is the dilution used in STs. In a previous article by our group (Torres et al., 2012), we found a higher sensitivity for IDT using undiluted ICMs than 10-fold diluted ICM with 100% specificity. Moreover, no patient with negative IDT had a positive patch test. This is the reason why we did not include patch test in the allergological work-up for this study.

It has been proposed that STs should be performed with a wide panel of different ICMs in order to identify tolerated alternative ICM (Vernassiere et al., 2004; Kvedariene et al., 2006; Caimmi et al., 2010; Torres et al., 2012; Yoon et al., 2015; Gracia-Bara et al., 2019; Brockow, 2020), mainly when the culprit is unknown. However, choosing non–cross-reactive ICM basing only on a negative ST could not completely prevent the recurrence of HSR, as in our study, 55% of patients reacted in DPT despite being negative in STs, what it is in line with previous data (Vernassiere et al., 2004; Torres et al., 2012). Moreover, in the group of patients allergic to multiple ICMs with a positive ST, tolerance to a negative-ST ICM could not be guaranteed, as DPT was positive in almost 50% of cases. Therefore, DPT should be considered not only to establish the diagnosis but also to choose the alternative even if STs are negative. The underlying mechanism of HSRs to ICMs is not well known, mainly in those cases with negative STs and positive DPT, and there may be a non-immune mediated mechanism involved. However, previous evidence supports an underlying immune mechanism in these reactions. In this sense, positive results in basophil activation test in patients with IRs and negative STs and positive DPTs to ICMs (Salas et al., 2013), indicate that an IgE-mechanism may be involved in IRs to ICM. Regarding NIRs, it has been previously demonstrated similar results in skin biopsies obtained from positive IDTs and DPTs (Torres et al., 2012), supporting a T cell involvement.

The most frequent cross-reactivity associations detected in our study were iodixanol and iomeprol, and iodixanol and iohexol. This pattern agrees with other reports (Vernassiere et al., 2004; Brockow et al., 2009; Hasdenteufel et al., 2011; Torres et al., 2012; Gracia-Bara et al., 2019). Mechanisms underlying the cross-reactivities between ICMs are not fully understood and further studies are necessary. Cross-reactivity has been related to the chemical structure (Vernassiere et al., 2004; Hasdenteufel et al., 2011; Lerondeau et al., 2016). ICMs are monomeric or dimeric derivatives of triiodobenzoic acid, with different organic side chains attached to the central benzene ring shared by all ICMs (Lerondeau et al., 2016). According to their chemical structure, four groups have been described: ionic tri-iodized monomers, ionic hexa-iodized dimers, nonionic tri-iodized monomers, and nonionic hexaiodized dimers. It has been reported a higher cross-reactivity between ICMs from the same group and a lower one between ICMs from different groups (Hasdenteufel et al., 2011). Such a high cross-reactivity in NIRs has been proposed to be attributed to nonspecific stimulation or pharmacological interaction with immune receptors across ICM. The presence of T cell clones has been demonstrated in previous studies (Lerch et al., 2007) along with specific recognition of the ICM in T cell receptors (Keller et al., 2010). In fact, it has been reported that iobitridol shows low cross-reactivity, mainly in patients with NIRs. The results of an *in vitro* test of T cell clones have shown that iobitridol is the least stimulatory ICM (Lerch et al., 2007). In our study, the ICM that less frequently induced reactions in DPT were iobitridol and iohexol in IRs and iomeprol in NIRs. This difference compared with published data may be related to a bias in our study as we could not performed DPT with all ICMs in all patients. Nevertheless, our aim was to describe the clinical characteristics of a series of patients allergic to multiple ICMs and the role of the different methods used for their diagnosis in real allergological practice.

Summarizing, this study has investigated HSRs to multiple ICMs. It shows that allergy to multiple ICMs is common, associated to severe reactions in IRs and confirmed frequently by positive STs. However, even in the case of negative-ST results, tolerance to an alternative ICM (including structurally unrelated ones) must be proven by DPT, as ST sensitivity is not sufficient. Therefore, DPT is necessary not only to confirm the diagnosis but also to identify safe alternative ICM before radiological examination.

## Data Availability Statement

The raw data supporting the conclusions of this article will be made available by the authors, without undue reservation, to any qualified researcher.

## Ethics Statement

The studies involving human participants were reviewed and approved by Comité de Ética de la Investigación Provincial de Málaga. The patients/participants provided their written informed consent to participate in this study.

## Author Contributions

ID, GB, MS, AT, and MT recruited patients and performed the clinical evaluations. ID, MT, JL, and EM contributed to study design. ID and MT wrote the first draft of the manuscript. ID, MT, JL, and EM corrected the manuscript. All authors contributed to the article and approved the submitted version.

## Funding

This work was supported by grants co-funded by the European Regional Development Fund (ERDF), from the Carlos III National Health Institute (ARADyAL network RD16/0006/0001, RD16/0006/0019, and RD16/0006/0033).

## Conflict of Interest

The authors declare that the research was conducted in the absence of any commercial or financial relationships that could be construed as a potential conflict of interest.

The handling editor is currently co-organizing a Research Topic with one of the authors MT, and confirms the absence of any other collaboration.
